# Exercise and nutrition routine improving cancer health (ENRICH): The protocol for a randomized efficacy trial of a nutrition and physical activity program for adult cancer survivors and carers

**DOI:** 10.1186/1471-2458-11-236

**Published:** 2011-04-15

**Authors:** Erica L James, Fiona Stacey, Kathy Chapman, David R Lubans, Gabrielle Asprey, Kendra Sundquist, Allison Boyes, Afaf Girgis

**Affiliations:** 1 School of Medicine and Public Health, University of Newcastle, Hunter Medical Research Institute, Priority Research Centre for Health Behaviour, Priority Research Centre in Physical Activity and Nutrition, Callaghan NSW Australia; 2 Cancer Council NSW, Woolloomooloo NSW Australia; 3 School of Education and Priority Research Centre in Physical Activity and Nutrition, University of Newcastle, Callaghan NSW Australia; 4 Centre for Health Research and Psycho-oncology (CHeRP), University of Newcastle and Cancer Council NSW, Hunter Medical Research Institute, and Priority Research Centre for Health Behaviour, Callaghan NSW Australia

## Abstract

**Background:**

The Exercise and Nutrition Routine Improving Cancer Health (ENRICH) study is investigating a novel lifestyle intervention aimed at improving the health behaviors of adult cancer survivors and their carers. The main purpose of the study is to determine the efficacy of lifestyle education and skill development delivered via group-based sessions on the physical activity and dietary behaviors of participants. This article describes the intervention development, study design, and participant recruitment.

**Methods/Design:**

ENRICH is a randomized controlled trial, conducted in Australia, with two arms: an intervention group participating in six, two-hour face-to-face sessions held over eight weeks, and a wait-list control group. Intervention sessions are co-facilitated by an exercise physiologist and dietician. Content includes healthy eating education, and a home-based walking (utilizing a pedometer) and resistance training program (utilizing elastic tubing resistance devices). The program was developed with reference to social cognitive theory and chronic disease self-management models. The study population consists of cancer survivors (post active-treatment) and their carers recruited through community-based advertising and referral from health professionals. The primary outcome is seven-days of sealed pedometry. Secondary outcomes include: self-reported physical activity levels, dietary intake, sedentary behavior, waist circumference, body mass index, quality of life, and perceived social support. The outcomes will be measured at baseline (one week prior to attending the program), eight-weeks (at completion of intervention sessions), and 20-weeks. The intervention group will also be invited to complete 12-month follow-up data collection. Process evaluation data will be obtained from participants by questionnaire and attendance records.

**Discussion:**

No trials are yet available that have evaluated the efficacy of group-based lifestyle education and skill development amongst mixed groups of cancer survivors and their carers. The results will have implications for the planning and provision of health and support services during the cancer survivorship phase.

**Clinical Trials Registration:**

Australian New Zealand Clinical Trials Register identifier: ANZCTRN12609001086257.

## Background

The number of cancer survivors worldwide is expected to triple from 25 million in 2008 to 75 million in 2030 [1]. There are approximately 340,000 cancer survivors in Australia, representing about 2% of the Australian population [2]. Cancer survivors are at increased risk of chronic illnesses such as cardiovascular disease and osteoporosis, death from non-cancer causes, cancer recurrence, secondary cancers, as well as long-term and/or late effects of treatment, such as fatigue, depression, pain, reduced quality of life (QoL), and weight loss or gain [3-5]. These increased risks can be attributed to cancer treatment, genetic predisposition, and common lifestyle factors [6].

The role of lifestyle factors such as nutrition, physical activity (PA) and a healthy weight aimed at preventing recurrence, secondary cancers, and other chronic diseases is an emerging area of research [7,8]. The benefits of PA for people affected by cancer include improved cardiovascular fitness, modest improvements in reducing fatigue, and improved mood and quality of life, body composition, sleep, self-esteem, depression, anxiety, and tiredness [9,10]. In breast and bowel cancer survivors, PA is associated with lower risk of disease recurrence and longer survival [11-13]. Resistance training can be safely performed [14], and has been associated with improvements to self esteem, muscular strength, and lean body mass [15]. Evidence is emerging that the pattern of activity is important, with unique metabolic consequences associated with prolonged sedentary behavior [16]. There is a dose-response association between sitting time and mortality from all causes, that is independent of leisure time activity [17], and is associated with the development of bowel, endometrial, ovarian, and prostate cancer, and cancer-specific mortality in women [18].

Diet quality after a breast cancer diagnosis is directly associated with subsequent mental and physical functioning [19,20]. Dietary fat reduction and modest weight loss has also been associated with relapse-free survival in post-menopausal breast cancer patients [21]. Both diet and PA contribute to the development of obesity, itself an independent contributor to risk of cancer recurrence and survival [7,22]. Evidence suggests that making changes in health behaviors (healthy diet and PA) after a cancer diagnosis may have a significant impact on health [23].

This increasing importance of nutrition and PA for cancer survivors, has been recognized in recent guidelines. An international review by the World Cancer Research Fund and American Institute for Cancer Research concluded that cancer survivors should follow the same diet, healthy weight, and physical activity principles for cancer prevention as the general population [7]. These recommendations are: to be as lean as possible within a healthy body weight; be physically active; limit energy dense food and drink; eat mostly foods of plant origin; limit red meat and avoid processed meat; limit salt; and aim to meet nutritional needs through diet alone [6]. The American College of Sports Medicine and Exercise and Sports Science Australia both acknowledge the safety and efficacy of exercise training for cancer survivors, with general recommendations of low to moderate intensity, three to five times per week, and involving aerobic, resistance, or mixed exercise types [14,24].

Despite these lifestyle recommendations from key agencies and professional organizations, cancer survivors' lifestyle behaviors are similar to the general population. An Australian study of cancer survivors found that unhealthy behaviors (physical inactivity, low fruit and vegetable consumption, overweight/obesity, high alcohol consumption) were similar, if not worse, than a matched sample of persons without a cancer history and survivors were also more likely to report a range of chronic co-morbid conditions [25]. Cancer survivors report being interested in lifestyle behaviors, with some evidence suggesting that a cancer diagnosis may provide a 'teachable moment' to improve health [26].

Little is known about the lifestyle behaviors of the carers of cancer survivors. An Australian study reported that for people with a friend or relative diagnosed with cancer, the diagnosis may have been a cue to make positive diet improvements and increase PA [27]. However, research suggests no difference between carers and non-carers on fruit and vegetable consumption, smoking status, alcohol consumption, PA, healthy body weight, or number or type of chronic illness [28,29]. Carers are likely to share many of the same behavioral risk factors as cancer survivors, and would benefit from improvements. Social support appears beneficial in helping cancer survivors make positive changes to exercise behavior [23]. Involving family members in health behavior interventions has positive effects on the patient's adherence to rehabilitation programs, and improved diet and PA behaviors [30,31].

Despite the growing evidence of the benefits and efficacy of lifestyle behaviors in promoting good health and recovery for cancer survivors, there are few services outside of the clinical setting specifically targeting cancer survivors to improve their health. Cancer survivors often report a sense of loss, and feeling "abandoned" or "cast adrift" by the health care system at the time of treatment completion [32]. There is a need to improve support and health services to assist those affected by cancer in the transition from patient to survivor. The main purpose of the present study is to evaluate the acceptability and efficacy of a healthy lifestyle (PA and diet) intervention for cancer survivors and their carers.

## Methods

### Study design

The study utilizes a two-arm randomized controlled trial design with a wait-list control group. Participants complete data collection at baseline, eight and 20 weeks. Intervention participants also complete these measures again at 12 months from baseline. The intervention consists of six, two-hour face-to-face group sessions held over eight weeks. Control group participants are invited to attend ENRICH after completion of the baseline, eight, and 20 week measures. Ethics approval was obtained from the University of Newcastle Human Research Ethics Committee (H-2009-0347). The design, conduct and reporting of this study will adhere to the Consolidated Standards of Reporting Trials (CONSORT) guidelines [33].

### Setting

Three intervention programs and three control programs have been conducted in Sydney, New South Wales, Australia. A further four to six programs are planned to occur in 2011.

### Participants

#### Selection criteria (eligibility)

Participation in the program is open to cancer survivors with a previous diagnosis of any type or stage of cancer and who had completed all active treatment (surgery, chemotherapy, radiotherapy, immunotherapy, bone marrow transplants, etc), and who do not have any food restrictions as a result of surgery or treatment, and to carers of cancer survivors. The inclusion criteria of the study are: 1) cancer survivor or carer of cancer survivor; 2) aged 18 years or older; 3) fluent in English; 4) signed medical clearance from their General Practitioner, and 5) with a functional performance score of two or less on the Eastern Cooperative Oncology Group criteria (that is "at least ambulatory and capable of all self-care but unable to carry out any work activities or up and about more than 50% of waking hours") [34]. Both cancer survivors and carers need to meet eligibility criteria. Survivors and carers may participate independently (that is, the survivor does not need a carer to participate and vice versa) or together. A survivor may also bring more than one carer.

#### Recruitment

Participants are recruited via multiple methods, including referrals from health professionals, medical centers, professional organizations (such as the Dieticians Association of Australia, New South Wales Oncology Groups), community health centers, cancer support groups, local media, and various Cancer Council NSW resources (website, mailing lists, and publications).

### Randomization

Stratified randomization by age group (less than 50, 50-65, older than 65) and gender is used with a block size of four. A random number sequence for each strata is generated using a random function in SAS version 9.2 and generated a random sequence of A and B's to indicate allocation to intervention or control groups.

The Project Co-ordinator uses a random number table to allocate consenting participants to intervention or control group, stratified by age and gender. If a participant has a partner or carer also consenting, they will be randomized together to the same group, stratified by age and gender of the cancer survivor. The Project Co-ordinator is not blinded to group allocation and participant blinding is not possible due to the wait-list control design of the trial.

### Statistical power and sample size

To detect a mean difference of 2000 steps per day in pedometer-derived step counts between the intervention and control groups with 80% power and 5% significance, assuming a standard deviation of 3200 steps, requires 42 subjects per group. The effect size estimate of 2000 steps per day change as the primary outcome is based on published research and clinically meaningful difference [35]. To ensure adequate sample size for secondary outcomes and to account for attrition and missing data, we aim to recruit 75 subjects per group.

### Outcomes

A pen-and-paper survey is completed at baseline (one week prior to first ENRICH program session), eight weeks (at the end of the ENRICH program), 20 weeks from baseline, and for the intervention group only, at 12 months from baseline. At each of these time-points, participants wear a sealed pedometer for seven days and complete a step count diary.

#### Primary outcome

The primary outcome is step counts as measured by seven days of sealed pedometry, that is used to provide an average daily step count [36]. Using a sealed pedometer prevents reactivity to monitoring step counts [37]. Despite variation in step counts on different days of the week, three days is the minimum amount of pedometer data required to estimate pedometer-determined PA in a week [38]. The step count diary allows self-report of instances where the pedometer was intentionally removed (eg. swimming, vigorous sports, sleeping) and when the participant forgot to wear the pedometer.

#### Secondary outcomes

##### Physical activity levels

PA frequency and duration is measured via eight items from the Active Australia survey. The survey evaluates four types of PA in the last week: brisk walking, moderate leisure activity, vigorous leisure activity, and vigorous household or garden chores [39].

Two items assessing the frequency and duration of resistance training were purpose-designed for this study and use the same wording and format of the Active Australia survey questions.

##### Sedentary behavior

Sedentary behavior is measured using a five-item question asking about time spent sitting (hours and minutes) during the last working and non-working day in each of the following domains: (a) while travelling to and from places (e.g., work, shops); (b) while at work; (c) while watching television; (d) while using a computer at home; and (e) at leisure not including watching television (e.g., visiting friends, movies, eating out) [40].

##### Dietary intake

Dietary intake is measured using a food frequency questionnaire, the Dietary Questionnaire for Epidemiological Studies (DQES version 2) [41]. The DQES version 2 contains a list of 74 items across four main categories (1. Cereal foods, sweets & snacks; 2. Dairy products, meat and fish; 3. Fruit; 4. Vegetables) with ten frequency response options ranging from 'Never' to 'three or more times per day'. It also contains three photographs of scaled portions for four foods (used to calculate a portion size calibrator); questions on the overall frequency of consumption of fruits and vegetables (used to calibrate the overestimation of these foods in the food list); and questions on consumption of foods such as bread that do not fit easily into the frequency format. Three questions covering alcohol consumption are also included as part of the DQES [41].

##### Waist circumference

Participants are asked to measure and report their own waist circumference, using standardized instructions [42]. Self-reported waist circumference has been reported as a satisfactory and reliable proxy for objective circumference measures [43].

##### Body mass index (BMI)

Participants report their height and weight, to allow calculation of body mass index. Data from an Australian study reports strong correlations between self-report and clinic measurements, with a trend for self-reported height to be over-estimated (especially in those aged 65 or older), and self-reported weight to be under-estimated [44].

##### Quality of life

Quality of life is assessed using the Medical Outcomes Study Short-Form-12 version 2: a 12-item measure evaluating quality of life across eight health domains: physical functioning (two items), role limitations due to physical health problems (two items), bodily pain (one item), general health perceptions (one item), vitality (energy/fatigue) (one item), social functioning (one item), role limitations because of emotional problems (two items), and general mental health (two items) [45]. These items can be used to calculate two subscales: physical and mental summary scales.

##### Social support

Social support is assessed using the 19-item Medical Outcomes Study Social Support Scale and assesses five domains of social support: informational support (four items), affection (three items), tangible support (four items), emotional support (four items), and positive social interaction (four items) [46].

##### Social cognitive mediators of physical activity

Hypothesized social cognitive mediators of PA behavior are assessed using existing validated scales. Due to a lack of validated measures for assessing mediators in relation to diet, these were not included in the survey.

An eight week time reference is provided for all the following social cognitive constructs. "Regular physical activity" is defined as *"achieving at least 30 minutes of moderate or vigorous-intensity activity on most, preferably all, days of the week" *which is consistent with national guidelines for Australian adults [47].

*Behavioral Goal *is assessed by asking participants on a scale of 0 per cent to 100 per cent, "How likely is it that you will do regular PA within the next eight weeks?" [48].

*Self-Efficacy *is measured with a nine-item scale [49]. Participants are asked to rate their confidence (*1 *= *not at all confident *to *5 *= *extremely confident*) that they could participate in regular PA over the next eight weeks when: a little tired; in a bad mood or feeling depressed; doing it by themselves; it became boring; there are no noticeable improvements in fitness; having other demands; feeling stiff or sore; there is bad weather; or having to get up early even on weekends.

*Outcome Expectations *are measured with five items [49]. The items in the scale assessed the extent to which individuals agree or disagree (*1 = strongly disagree *to *5 = strongly agree*) that participating in regular PA over the next eight weeks would for them: reduce tension or manage stress; feel more confident about one's health; sleep better; have a more positive outlook; or help control weight.

*Impediments *are measured with five items [49]. The items for this scale assess the extent to which individuals agree or disagree (*1 = strongly disagree *to *5 = strongly agree*) that participating in regular PA over the next eight weeks would for them: take too much of my time; have less time for my family and friends; make one too tired because of other daily responsibilities; make one worry about looking awkward if others saw them being physically active; or cost too much money.

*Social Support *is measured using a two-item scale [48]. Participants are asked whether over the next eight weeks people in their social network are likely to help them participate in regular PA, and whether they feel that someone in their social network will provide the support they need in order to be regularly physically active.

##### Demographics

The following demographic information is collected at baseline: gender, year of birth, postcode, marital status, education, current employment, current family income, and smoking status.

##### Health status

At baseline, participants are asked six questions about their health and service use (use of any nutrition and PA support service, diagnosed with chronic health problems, whether they are a cancer survivor, carer, or both, and if a carer, relationship to the cancer survivor), and four questions about cancer diagnosis (type of cancer, when diagnosed, treatments received, status of cancer).

##### Health Education Impact Questionnaire

Cancer survivors are asked to complete the 40-item Health Education Impact Questionnaire (heiQ). The heiQ provides a broad profile of the potential impacts of patient education programs [50] across eight domains: health directed behavior (four items); positive and active engagement in life (five items); emotional well-being (six items); self-monitoring and insight (six items); skill and technique acquisition (four items); constructive attitudes and approaches (five items); social integration and support (five items); and health services navigation (five items) [50].

### Process evaluation

Participant evaluation of the ENRICH program and sessions is measured via a participant program evaluation form (to be completed by participants at the final ENRICH session), and audit of participant attendance lists. The program evaluation form consists of nine questions from the course evaluation module of the heiQ [51]. A further eleven questions evaluating the program were purpose-designed by the research team.

ENRICH program facilitators are asked to provide their feedback on each of the sessions via pen-and-paper form. This enables the facilitators to reflect on any aspects of the program content that did or didn't work; any particular issues not well addressed; and whether or not there were any issues affecting the group sessions.

### Procedure

**Figure 1:**
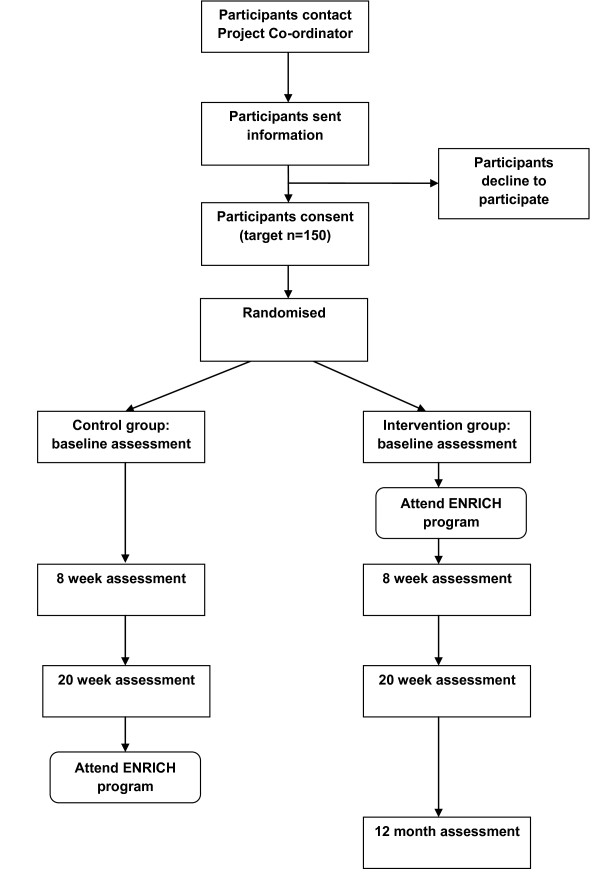
**Study flow**.

Participants are asked to complete the pen-and-paper survey, wear their pedometer for seven-days and complete a pen-and-paper step count diary at baseline, eight, and twenty weeks from baseline (see Figure 1). In addition, the intervention group will also complete these measures at 12 months from baseline. Participants who do not return their survey, diary, and pedometer within two weeks receive one reminder call from the Project Co-ordinator. If these materials are not returned after a further two weeks, the Project Co-ordinator phones them to conduct a second and final reminder call.

Participants are asked to attend each of the six ENRICH program sessions. Attendance records are maintained by the Project Co-ordinator.

### Intervention development and content

ENRICH consists of four weekly sessions, followed by two fortnightly sessions. Each session consists of a mix of healthy eating information and activities, a home-based walking program information and resources (with pedometer), and a home-based resistance training program (with elastic tubing equipment, Gymstick™) with information and resources.

ENRICH program sessions are co-facilitated by an exercise physiologist (exercise specialist) and dietician who received training from members of the research team. Educational information is supplemented with practical activities (eg. food label reading), group discussion (eg. brainstorming ideas to overcome barriers to being more active), and role modelling and practice of resistance training exercises, including stretching and appropriate warm-ups and cool down. The practice is to assist participants to become familiar and comfortable with the equipment, and allows the exercise physiologist an opportunity to ensure correct technique. Participants receive a pedometer, Gymstick™, step count diary, and written resources as part of the program.

A Gymstick™ http://www.gymstick.net is a lightweight graphite shaft, with elastic tubing and foot straps that provide resistance and can be used to exercise all of the major muscle groups. It is a safe, effective and relatively inexpensive tool for a strength-based exercise program to be conducted at home [52]. Gymsticks™ are available in five different resistance levels and the load on each device can be increased by rolling the bar and shortening the elastic tubing [52].

The program content was developed by experts in each of the content areas (members of research team), with input from advisory and working group members, and reviewed by oncology dieticians and physiotherapists. The program content is structured, but allows flexibility in the delivery to allow for discussion, questions, and interactive activities.

The ENRICH program content and delivery approach was guided by Bandura's (2004) Social Cognitive Theory [53] and a chronic disease self-management approach [54]. Examples of social cognitive theory program constructs include: building self-efficacy (self-monitoring of behavior through use of step count diaries); identification of socio-cultural factors such as facilitators and impediments (brainstorming barriers and strategies to exercise when the weather is bad); outcome expectations (use and reflection of step count diaries); goal setting (setting and monitoring step count goal); and knowledge of risks and benefits (of PA, healthy eating and healthy weight management).

Traditional chronic disease self-management models encourage participants to take responsibility for their own health and behavior to make sustainable, life-long changes [54]. Chronic disease self-management has similar values to the core social cognitive theory constructs with key elements of chronic disease self-management being self-efficacy, motivation, developing achievable action plans, and role modelling [54].

### Statistical methods

Data will be entered into SAS version 9.2. Initial descriptive analyses will be used to describe the socio-demographic and disease characteristics of the participants. Independent t-tests (or other non-parametric equivalents) will be used to assess difference between the intervention and control groups. Paired t-tests (or other non-parametric equivalents) will be used to assess changes in outcomes from baseline to follow-up. Longitudinal data will be analyzed using generalized estimating equation (GEE) models. Analyses will take into account potential clustering of dyad behaviors. Potential mediators of PA behavior change will be assessed using a product-of-coefficients test.

## Discussion

The ENRICH program meets a current gap in the provision of care to cancer survivors and carers, during the survivorship phase. The program focuses on important lifestyle behaviors that have the potential to address long-term and late effects of cancer and treatment, as well as prevention of other chronic health conditions. The program was intentionally developed to be applicable to cancer survivors diagnosed with different cancers and at different stages. The program has the ability to be individually tailored, and because it is not prescriptive, is relevant to all group members. The focus of the program is on giving participants the skills and knowledge to make achievable, life-long change appropriate to the ability of participants.

Limitations of the study include the reliance on self-report data for body composition and dietary behavior. It was not possible to obtain objective waist and weight measures from the control group participants, without potentially influencing their behaviors during the intervention period.

A novel approach of the program is the emphasis on self-management constructs, and provision of materials for a predominantly home-based PA program. Home-based activity programs place control back with participants who can exercise at a pace and ability at their own comfort level. The use of a home-based resistance training device is also a novel aspect of the program, with the efficacy of an elastic tubing device yet to be demonstrated in this population. As much as ENRICH is designed for participants to implement themselves at home, there is further potential for this program to be modified and tested in different formats that might be applicable to those disadvantaged through distance, isolation, or transport.

## Conclusion

With increasing numbers of cancer survivors at-risk for long-term and/or late effects of treatment and other chronic disease, efforts for promoting the health of this important group are urgently needed. This lifestyle program may provide valuable information relating to the development of other healthy lifestyle interventions for cancer survivors and carers, and result in appropriate behavior change and self-management strategies.

## List of abbreviations used

ENRICH: Exercise and Nutrition Routine Improving Cancer Health; PA: Physical activity; QoL: Quality of life; DQES: Dietary Questionnaire for Epidemiological Studies; BMI: Body mass index; heiQ: Health Education Impact Questionnaire; GEE: Generalized Estimating Equation.

## Competing interests

The authors declare that they have no competing interests.

## Authors' contributions

EJ, KC, and KS conceived the study and obtained the funding. All authors provided input into the study and intervention design. GA and FS were primarily responsible for recruitment and data collection. FS, EJ, KC, and DL were responsible for drafting the manuscript. All authors critically evaluated the article for content and approved the final version.

## Pre-publication history

The pre-publication history for this paper can be accessed here:

http://www.biomedcentral.com/1471-2458/11/236/prepub
